# An Alternative Source for Allogeneic CAR T Cells With a High Safety Profile

**DOI:** 10.3389/fimmu.2022.913123

**Published:** 2022-05-23

**Authors:** Xiaolong Wu, Ingo G. H. Schmidt-Wolf

**Affiliations:** ^1^ Clinical Trial Center, National Medical Products Administration Key Laboratory for Clinical Research and Evaluation of Innovative Drugs, West China Hospital, Sichuan University, Chengdu, China; ^2^ Department of Integrated Oncology, Center of Integrated Oncology (CIO) Bonn, University Hospital Bonn, Bonn, Germany

**Keywords:** CIK (cytokine-induced killer) cells, CAR (chimeric antigen receptor) T cells, allogeneic, safety, efficacy

## Introduction

Chimeric antigen receptor (CAR) cell-based immunotherapy has emerged as a promising regimen for the treatment of patients with hematological malignancies. To date, five CAR T-cell therapies have been approved by the US Food and Drug Administration (FDA) (1), four of which target CD19-positive B-cell leukemia and lymphoma, and one targets B-cell maturation antigen (BCMA) expressing multiple myeloma. Despite excellent responses, there are still some common limitations of these approved products for widespread clinical application: (1) the generation of an autologous product for each individual patient is logistically cumbersome, (2) it is not always possible to generate clinically relevant doses of CAR T cells from heavily pre-treated patients, and (3) the high rate of higher-grade or lethal cytokine release syndrome (CRS) and neurotoxicity (2). In addition, one rare case was reported in which tumor cell-based contamination during the manufacturing process can lead to relapse due to accidental transduction with a CAR (3). Therefore, donor-derived T cells with a high safety profile are required to overcome these obstacles.

Cytokine-induced killer (CIK) cells are a heterogeneous population, consisting of NK, NK-T, and T cells, generated by culturing peripheral blood mononuclear cells (PBMCs) in the presence of INF-gamma, anti-CD3 antibody, and IL-2 for 2–3 weeks (4). Mature CIK cells typically consist of more than 95% of CD3 T cells with majority of cells expressing CD8 and CD56. Sharing phenotypical and functional characteristics of T and NK cells, CIK cells are endowed with the ability to recognize and eliminate tumor targets in both MHC-dependent and -independent manners (5). Previous clinical data have shown a favorable safety profile of donor-derived CIK cell therapy (6), with low incidence of graft-versus-host disease (GVHD), which suggests a suitable candidate for CAR-based cell therapy.

## The First-in-Human Clinical Study of CAR CIK Cells

### Efficacy

Recently, Magnani et al. (7) conducted the first-in-human clinical trial using donor-derived CD19-CAR CIK cells for treatment of relapsed B-ALL patients after allo-hematopoietic stem-cell transplantation (HSCT). The outcome was very encouraging, showing that six out of 7 patients receiving the highest doses of CD19-CAR CIK cells achieved complete response (CR) and CR with incomplete blood count recovery (CRi) at day 28. Five out of 6 patients in CR were also minimal residual disease-negative, four of whom remained in remission with a median follow-up of 6.9 months as of the data cutoff date. B-cell aplasia was sustained at the last follow-up in 6 out of 13 patients, with a median duration of 3 months. Despite the promising outcomes, whether CD19-CAR CIK cells have comparable durable response to current commercially available CD19-CAR T-cell products remains to be determined by evidence from more clinical trials with a large cohort of patients and long-term follow-up. Some clinical data have identified that responses correlate with the expansion, persistence, and a memory phenotype of CAR T cells (8, 9). In this study (7), the median time to peak expansion of CD19-CAR CIK cells was 14 days, akin to those in conventional CD19-CAR T trials, as measured by transgene copy. However, the majority of CD3+ CAR CIK cells had a CD8+ effector memory phenotype. It has been reported that despite CD8+ effector memory T cells having strong cytotoxic effector memory properties, only central memory T cells and other less differentiated T-cell subsets, such as naive T cells and stem cell-like memory T cells, are critical for *in vivo* expansion, survival, and long-term persistence (10). Therefore, less differentiated CAR CIK cells may lead to better antitumor response.

Cytokines are essential for expansion and persistence of CIK cells. It was reported that IL-15-activated CIK cells have an increased anti-leukemic potential *in vitro* compared to conventional IL-2-activated CIK cells (11). However, systemic administration of IL-15 in patients can lead to severe toxicities. Transduction of a gene for autocrine production of IL-15 by CIK cells themselves appears to be an appreciable option, supporting the transferred CAR CIK cells in recipients without inducing severe side effects.

### CD3+CD56- CIK Subset Might Be the Dominant Component of CAR CIK Effector Cells

Most previous studies investigated the bulk CIK cells re-engineered with a CAR structure (12, 13). Although *in vitro* data have shown superior antitumor effect of unmodified CD3+CD56+ CIK cells over the CD3+CD56- counterpart in the context of non-MHC restriction (14, 15), this difference might not be obvious when CIK cells are genetically introduced with a CAR component. Both subsets have high levels of T-cell receptor (TCR) downstream signaling molecules in places that can be recruited by CAR signaling. Furthermore, since the CD3+CD56- CIK subset is the precursor of CD3+CD56+ CIK cells and possesses higher proliferative capacity (16), a better *in vivo* antitumor efficacy can be assumed after CAR gene transduction. One early study showed that CD28-ζ–OX40 CAR accelerated terminal maturation of CD56+ CIK cells, which were prone to activation-induced cell death (AICD) and reduced antitumor efficiency *in vivo* compared to CD28-ζ CAR (17). Moreover, they demonstrated that CAR-redirected CD3+CD56+ CIK cells were less efficient than their CD3+CD56- counterpart in tumor elimination *in vivo* irrespective of the CAR-provided costimulation. As discussed (17), the result was partially explained by the susceptibility of CD3+CD56+ CIK cells to AICD. Another possibility, as we speculate, is due to the higher percentage of the CD3+CD56- subset presenting in CD28-ζ CAR CIK cells, which possesses similar antitumor efficiency but with greater proliferative index in comparison to the CD3+CD56+ subset. In this report (7), the investigators introduced a CD28-ζ–OX40 CAR, which was previously shown to promote generation of the CD56+ phenotype by the same group (18). In contrast, CD28-ζ CAR led to no alteration on the development of CIK subsets as reported by others (19). Since no real clinical data with CD28-ζ CAR CIK cells are available, it is difficult to draw a conclusion on which CAR construct is optimal for CIK cell-based therapy. The currently FDA-approved CAR T products all exploit a second generation of CAR construct incorporated with either CD28 or 4-1BB as a costimulatory domain. Therefore, it would be reasonable to test these in future CAR CIK cell studies.

In studies with unmodified CIK cells, higher percentage of CD3+CD56+ cells are typically considered as a major contributor to antitumor efficacy. By contrast, in the scenario of CAR-based cell therapy, more benefit might be achieved when the focus is skewed to the CD3+CD56- CIK subset. One way to obtain more CD3+CD56- CIK cells is by shortening the *ex vivo* expansion time to 10–14 days instead of 20 or 30 days. In this clinical trial (7), however, the cell product manufacturing was prolonged to a median length of 23 days (range, 20–32 days) in order to reach a complete clearance of SB11 transposon activity, viewed as a potential weakness of this study by the authors. They also proposed alternative platforms (using the hyperactive SB100X variant in mRNA or protein version) to mitigate this weakness. In addition, transduction with viral vectors may also narrow the expansion period of CAR CIK cells.

### Safety

More impressively, no GVHD and neurotoxicity were observed in this study (7), with only grade 2 or grade 1 CRS. Consistent with early clinical data (6), CAR CIK cells generated from HLA-matched unrelated or haploidentical donors were safe and tolerable in recipients. The mechanism by which allogeneic CIK cells lead to minimal GVHD remains unclear. An early study showed that the high level of IFN-gamma production by CIK cells contributed to this minor GVHD (20). The limited *in vivo* proliferative ability of CIK cells, especially the CD3+CD56+ subpopulation, may have also made a contribution. Nevertheless, this unique attribute renders CIK cells as a safe and promising source for new biotechnological investigation in adoptive immunotherapy, including the CAR-based cell therapy. The starting dosage of CD3+CD56- CAR T cells in this trial was comparable to those used in conventional CAR T-cell studies (2–7.5 × 10^6^ cells/kg), as the CD3+CD56- subset accounted for nearly half of CAR CIK cells (up to 15 × 10^6^ bulk cells/kg) (7), further suggesting that CAR T cells cultured under this condition were well tolerated with low risk of GVHD occurrence.

### Other Strategies or Sources for Generation of Off-the-Shelf CAR T Cells

Recently, other strategies are being tested to generate allogeneic CAR T cells as an off-the-shelf product, mostly by knocking out the gene encoding TCR and/or disrupting HLA molecules (β2-microglobulin) on donor T cells to prevent the GVHD and/or the transplant rejection, showing encouraging response with no GVHD (21). However, a major pitfall of TCR knockout is the deprivation of natural antitumor effect mediated by the endogenous TCR system of CAR T cells, which may play a role in the long-term control of disease. It remains unknown how TCR disruption impacts *in vivo* T-cell proliferation in this context. One recent study showed that endogenous TCR promotes *in vivo* persistence of CD19-CAR T cells compared to a TCR knockout CAR (22). In addition, multigene manipulation needs more advanced technologies with higher cost and might increase the likelihood of chromosomal aberration of the transferred CAR T cells (23). Apart from the gene-edited TCR knockout, another new method has been recently developed by the introduction of the anti-CD3ε protein expression blocker (PEBL), which led to intracellular retention of CD3ε and, in turn, prevented expression of TCRαβ on the surface of T lymphocytes (24). Unlike the gene-editing system, this approach would not pose new concerns over the chromosomal aberration, but with full efficiency in blocking the endogenous TCR pathway. However, the efficacy and safety of allogeneic CAR T cells engineered by the PEBL system remains to be determined in future clinical studies.

Furthermore, other sources including γδ T cells and NKT cells are also being investigated for the production of allo-CAR T cells. There are few ongoing clinical trials testing CAR-γδ T cells, but none has yet to release their data. On the basis of preclinical data, γδ T cells seem to be a good source for the generation of the off-the-shelf CAR cell product, as they are capable of effectively restraining tumor growth in multiple mouse models (25, 26). Recently, the interim analysis from two clinical studies using CAR NKT cells has been reported, showing a favorable safety profile of CAR NKT cells with early evidence of antitumor response (27, 28). Since only limited clinical data have been reported on these two sources and CIK cells in CAR-based settings, it is too early to make comparisons between them. Notwithstanding, in terms of product manufacturing, CIK cells appear to have advantages. Although purification during or after expansion of CAR γδ T cells or CAR NKT cells is required to avoid any T-cell contamination in the final product, it is not necessary in the case of CAR CIK cell production. In addition, it is not always able to generate a clinical relevant dose of CAR NKT cells, due to the paucity of NKT cells in human PBMCs (~0.1%). Moreover, CIK cells still retain their TCR machinery, which allows them to eradicate target cells or proliferate in an MHC-dependent manner, but not causing severe GVHD as conventional donor-derived T cells do.

Overall, among these potential alternatives, CIK cells hold a great position to be a suitable source for allogeneic CAR T-cell therapy, with the following advantages: (1) convenient and massive expansion capability, (2) minimal to no GVHD and low-grade side effects (CRS and neurotoxicity), and (3) the ability to attack targets through its own intact TCR repertoire.

## Concluding Remarks

**Figure 1:**
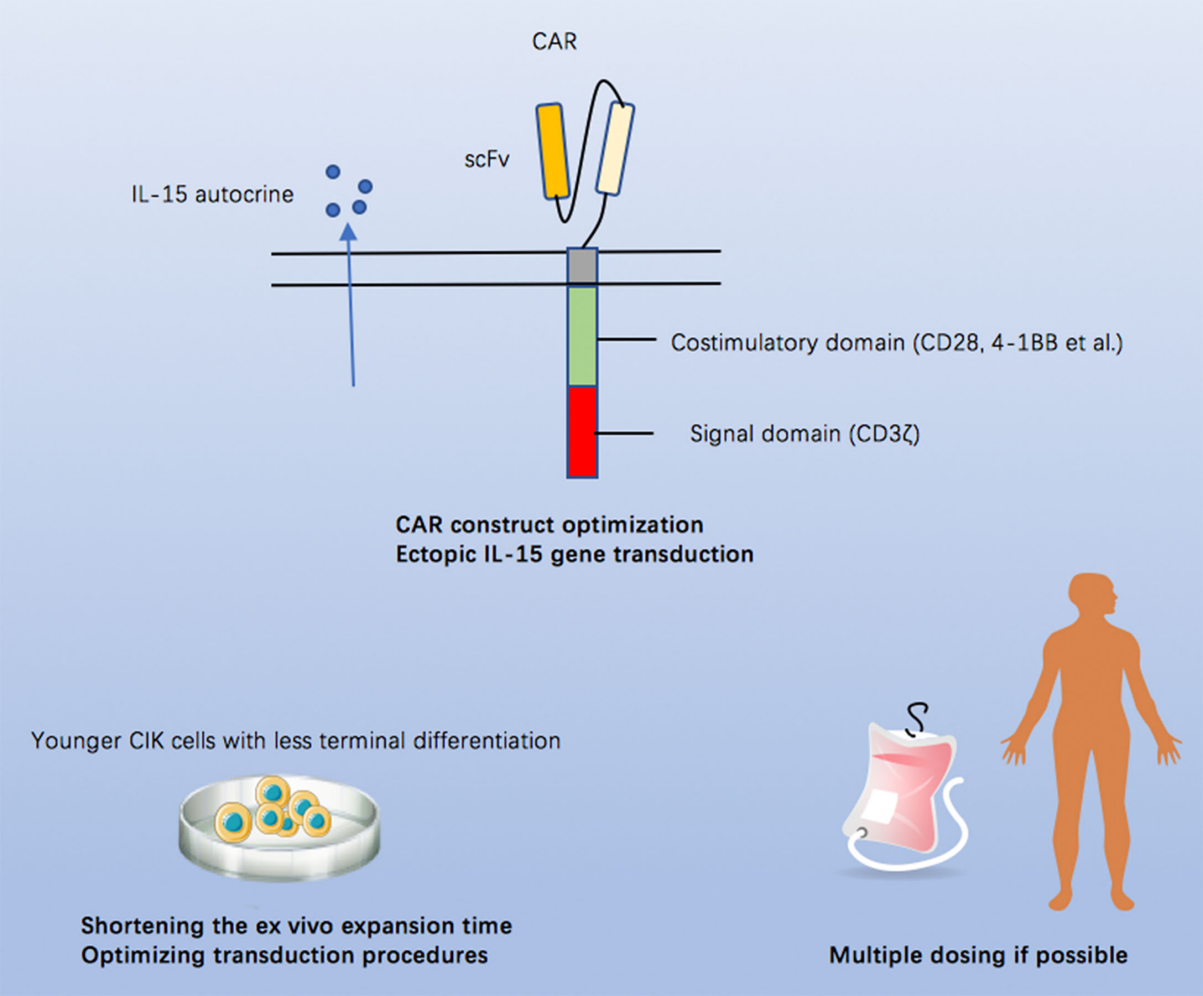
Possible approaches to improve the potency or efficacy of CAR CIK cells.

The first-in-human CAR CIK cell trial provided us early impressive evidence demonstrating the feasibility, safety, and efficacy of this product and paving the way for further investigations. Here, we propose possible perspectives to improve the potency or efficacy of CAR CIK cells (**Figure 1**): (1) optimizing the CAR construct and/or incorporating an ectopic IL-15 gene to improve the antitumor capacity and persistence of CIK cells, (2) shortening the *ex vivo* expansion course to prevent CIK cells from becoming terminally differentiated or exhausted, and (3) multi-dosing if possible.

## Author Contributions

Writing—original draft: XW and IS-W. All authors contributed to the article and approved the submitted version.

## Funding

The CIO Aachen Bonn Köln Düsseldorf is kindly supported by the Deutsche Krebshilfe.

## Conflict of Interest

The authors declare that the research was conducted in the absence of any commercial or financial relationships that could be construed as a potential conflict of interest.

## Publisher’s Note

All claims expressed in this article are solely those of the authors and do not necessarily represent those of their affiliated organizations, or those of the publisher, the editors and the reviewers. Any product that may be evaluated in this article, or claim that may be made by its manufacturer, is not guaranteed or endorsed by the publisher.
